# Acute hypoxaemic respiratory failure in a low-income country: a prospective observational study of hospital prevalence and mortality

**DOI:** 10.1136/bmjresp-2020-000719

**Published:** 2020-11-04

**Authors:** Arthur Kwizera, Jane Nakibuuka, Lydia Nakiyingi, Cornelius Sendagire, Janat Tumukunde, Catherine Katabira, Ronald Ssenyonga, Noah Kiwanuka, David Patrick Kateete, Moses Joloba, Daphne Kabatoro, Diana Atwine, Charlotte Summers

**Affiliations:** 1 Anaesthesia and Critical Care, Makerere University College of Health Sciences, Kampala, Uganda; 2 Intensive Care, Mulago National Referral Hospital, Kampala, Uganda; 3 Internal Medicine, Makerere University Faculty of Medicine, Kampala, Uganda; 4 Respiratory medicine department, Royal Papworth Hospital NHS Foundation Trust, Cambridge, United Kingdom; 5 School of Public Health, Makerere University College of Health Sciences, Kampala, Uganda; 6 Immunology and Molecular Biology, Makerere University College of Health Sciences, Kampala, Uganda; 7 Office of the permanent secretary, Republic of Uganda Ministry of Health, Kampala, Uganda; 8 Dept of Medicine, University of Cambridge, Cambridge, UK

**Keywords:** clinical epidemiology, respiratory infection, ARDS

## Abstract

**Introduction:**

Limited data exist on the epidemiology of acute hypoxaemic respiratory failure (AHRF) in low-income countries (LICs). We sought to determine the prevalence of AHRF in critically ill adult patients admitted to a Ugandan tertiary referral hospital; determine clinical and treatment characteristics as well as assess factors associated with mortality.

**Materials and methods:**

We conducted a prospective observational study at the Mulago National Referral and Teaching Hospital in Uganda. Critically ill adults who were hospitalised at the emergency department and met the criteria for AHRF (acute shortness of breath for less than a week) were enrolled and followed up for 90 days. Multivariable analyses were conducted to determine the risk factors for death.

**Results:**

A total of 7300 patients was screened. Of these, 327 (4.5%) presented with AHRF. The majority (60 %) was male and the median age was 38 years (IQR 27–52). The mean plethysmographic oxygen saturation (SpO_2_) was 77.6% (SD 12.7); mean SpO_2_/FiO_2_ ratio 194 (SD 32) and the mean Lung Injury Prediction Score (LIPS) 6.7 (SD 0.8). Pneumonia (80%) was the most common diagnosis. Only 6% of the patients received mechanical ventilatory support. In-hospital mortality was 77% with an average length of hospital stay of 9.2 days (SD 7). At 90 days after enrolment, the mortality increased to 85%. Factors associated with mortality were severity of hypoxaemia (risk ratio (RR) 1.29 (95% CI 1.15 to 1.54), p=0.01); a high LIPS (RR 1.79 (95% CI 1.79 1.14 to 2.83), p=0.01); thrombocytopenia (RR 1.23 (95% CI 1.11 to 1.38), p=0.01); anaemia (RR 1.15 (95% CI 1.01 to 1.31), p=0.03); HIV co-infection (RR 0.84 (95% CI 0.72 to 0.97), p=0.019) and male gender (RR 1.15 (95% CI 1.01 to 1.31) p=0.04).

**Conclusions:**

The prevalence of AHRF among emergency department patients in a tertiary hospital in an LIC was low but was associated with very high mortality. Pneumonia was the most common cause of AHRF. Mortality was associated with higher severity of hypoxaemia, high LIPS, anaemia, HIV co-infection, thrombocytopenia and being male.

**Key messages**

- What is the prevalence of adult acute hypoxaemic respiratory failure in an acute care clinical setting in an African country and what are the survival outcomes of these patients?

- The mortality among adult patients presenting with AHRF in our study population was very high.

- This study provides the first direct evidence of the burden of AHRF in an African adult population presenting to a hospital emergency department and suggests that the high mortality may be associated with lack of access to advanced oxygen therapy as well as lack of access to ICU admission.

## Introduction

Acute hypoxaemic respiratory failure (AHRF) is a common cause of hospital admission and organ failure around the world.^1 2^ The severest form of AHRF is referred to as the acute respiratory distress syndrome (ARDS) and defined by the Berlin consensus (acute onset of hypoxaemia with PaO_2_/FiO_2_ ratio ≤300 mm Hg at a PEEP of 5 cm H_2_O, bilateral infiltrates on chest X-ray, with no need to exclude cardiac disease). Despite technological advances in mechanical respiratory support, the mortality of AHRF and ARDS remain high.^2 3^ Most of the published literature has focused on ARDS in the developed world.^4^ In a large Scandinavian study, the 90-day mortality rate in patients with AHRF was similar to that in ALI/ARDS, namely, 41% and 42%, respectively.^4^ In low-income countries (LICs) and especially Africa, where ICU capacities and resources are highly limited,^5^ there is paucity of data to describe the burden and epidemiology of AHRF or ARDS. Furthermore, the epidemiology of ARHF in LICs is poorly understood. One study conducted in Rwanda used a modification of the Berlin definition to determine the incidence of ARDS.^6^

We undertook this prospective study among medically ill adult patients presenting to the emergency department in a tertiary hospital in Kampala, Uganda, to determine the prevalence of and 90-day mortality from AHRF. Furthermore, we sought to determine risk factors associated with death in these patients.

## Methods

### Study design

This study was designed as a prospective, observational, period prevalence study and was conducted in the emergency department of the Mulago National Referral and Teaching Hospital in Kampala, Uganda, between 1 November 2015 and 31 July 2017. Written informed consent was obtained from all study patients or their next of kin.

### Patient and public involvement in the study

Patients and the public were not involved in the design, conduct, reporting or dissemination plans for this study.

### Study setting

Mulago National Referral and Teaching Hospital is a 1500-bed hospital located in Kampala, the capital city of Uganda. This hospital serves the population of Kampala, the capital city of Uganda, with 1.5 million inhabitants as well as the surrounding districts of Mukono and Wakiso with a total catchment population of 4 million people. The emergency department receives between 60 000 to 72 000 patients per year and is divided into three major areas namely a surgical side handling surgical emergency, a trauma resuscitation area and a medical side for medical emergencies. For purposes of our study we restricted ourselves to the medical side of the emergency department and focused on more severely ill patients with a Modified Early Warning Score >4.

A total of 13 nurses, 6 medical officers, 4 intern doctors and 1 full-time attending physician staffed the medical side of the emergency department. In addition, two senior house officers (internal medicine specialty trainees) and an on-call physician provide coverage on a rotational basis. The medical emergency ward was able to provide intermittent vitals monitoring as well as oxygen therapy. The emergency department did not provide non-invasive ventilation. In the emergency ward, patients spend 24 hours, during which a provisional/working diagnosis is initially made, and then after a diagnostic workup and emergency management, it may be amended at 24-hour review when a disposition plan is being made. Patients are then transferred to specialist units to receive further in-hospital care.

The hospital runs one of the two public ICUs in the country. During the study period, the ICU had 12 beds with the ability to ventilate six patients at any one time. It provides ICU services to all kinds of critically ill patients. The ICU can provide mechanical ventilation, postoperative care, intermittent haemodialysis, peritoneal dialysis and basic neurocritical care. Patients in the emergency and intensive care units can get full blood count and blood group tests done free of charge and chemistry panels at a cost.

### Study patients

All patients admitted to the ED with AHRF. AHRF was defined as acute shortness of breath of less than a week, with or without a history of cough, and a pulse oximetry reading of <91% on room air. We excluded patients who were younger than 18 years, patients with a poor prognosis, or patients who were unconscious and without a proxy to provide written informed or deferred consent.

### Informed consent

As far as possible, consent was obtained from patients. We obtained consent from the patients’ next of kin where the patient was too ill to give consent. In other cases, we obtained deferred consent, for patients who recovered within the study period. Additionally, due to the limited human resource in our setting, family members are often involved in in-hospital patient care and are therefore frequently available within hospital premises and are able to consent. Consent forms were translated into the local vernacular to aid communication.

### Medical management of study patients

On recruitment, all patients received continuous oxygen therapy by nasal prongs (6 L/min) or venturi masks (10 L/min) to provide at least FiO_2_ of 0.4 to maintain SpO_2_ levels above 91% or as best as possible. This population received treatment with conventional oxygen therapy (patients got oxygen therapy by face mask or nasal prongs with flow rates titrated against SPO_2_ >91% or as best as possible) and pneumonia management was in accordance with Ugandan national treatment guidelines (https://www.health.go.ug/sites/default/files/Uganda_Clinical_Guidelines_2016_FINAL.pdf). We referred patients who deteriorated further to the ICU as and when resources and bed capacity permitted.

Our study defined anaemia as haemoglobin <8 g/dL, thrombocytopenia as platelets <100×10^9^/L, leucocytosis as white cell count >12×10^9^/L and leucopenia as white cell count <4×10^9^/L. SPO_2_ <85% and mean arterial pressure <65 mm Hg were considered low.

### Data collection and processing

Using a standardised questionnaire, the study team obtained information on selected sociodemographic characteristics including age, gender, smoking status and firewood exposure. Additionally, clinical information such as previous health status and admission diagnosis as well as clinical and laboratory parameters (including predisposing conditions such as shock, sepsis, HIV status, pneumonia and risk modifiers such as need for higher FiO_2_, hypoxaemia) were obtained to compute the Lung Injury Prediction Score (LIPS). LIPS is a validated score in which one can incorporate a series of routinely available risk factors and modifiers to predict which patients will develop ARDS.^7^ In this model a LIPS of 4 predicts development of ARDS. Clinical notes were reviewed 24 hours after admission for change of or additional diagnoses.

In the absence of arterial blood gas results, we used the widely accepted and validated SpO_2_/FiO_2_ ratio to represent severity of hypoxaemia at admission. We used the SpO_2_/FiO_2_ ratio <315 as our threshold ratio for ARDS.^8^ In all patients, pulse oximetry was measured by conventional two-wavelength finger pulse oximeters. We considered the worst readable value of SpO_2_ at admission for our data.

At the end of each participant-researcher contact, case report forms were checked for completeness. We double entered the data into a computerised database (EpiData V.3.1)

### Study end points

The primary outcome was the prevalence of AHRF among study patients. The secondary outcome was 90-day mortality and factors associated with mortality.

### Statistical analysis

We conducted an 18-month period prevalence study. Prevalence of AHRF was computed using the total number of patients included as the denominator, and patients with AHRF as the numerator. Quantitative variables were described as mean (SD) or median (25th–75th centiles), as appropriate, and compared using Student’s t test or the Wilcoxon-Mann-Whitney test. Qualitative variables were described as counts (%) and compared using the χ^2^ or the Fisher’s exact test, as appropriate. The modified Poisson regression was used to determine the factors associated with mortality and this is expressed as risk ratios (RRs) with a two-sided p value level of significance set at 0.05. We included variables found to be associated with mortality in other acute care AHRF-related studies in our multivariate model, although they may not have been statistically significant at bivariate analysis.

## Results

**Figure 1:**
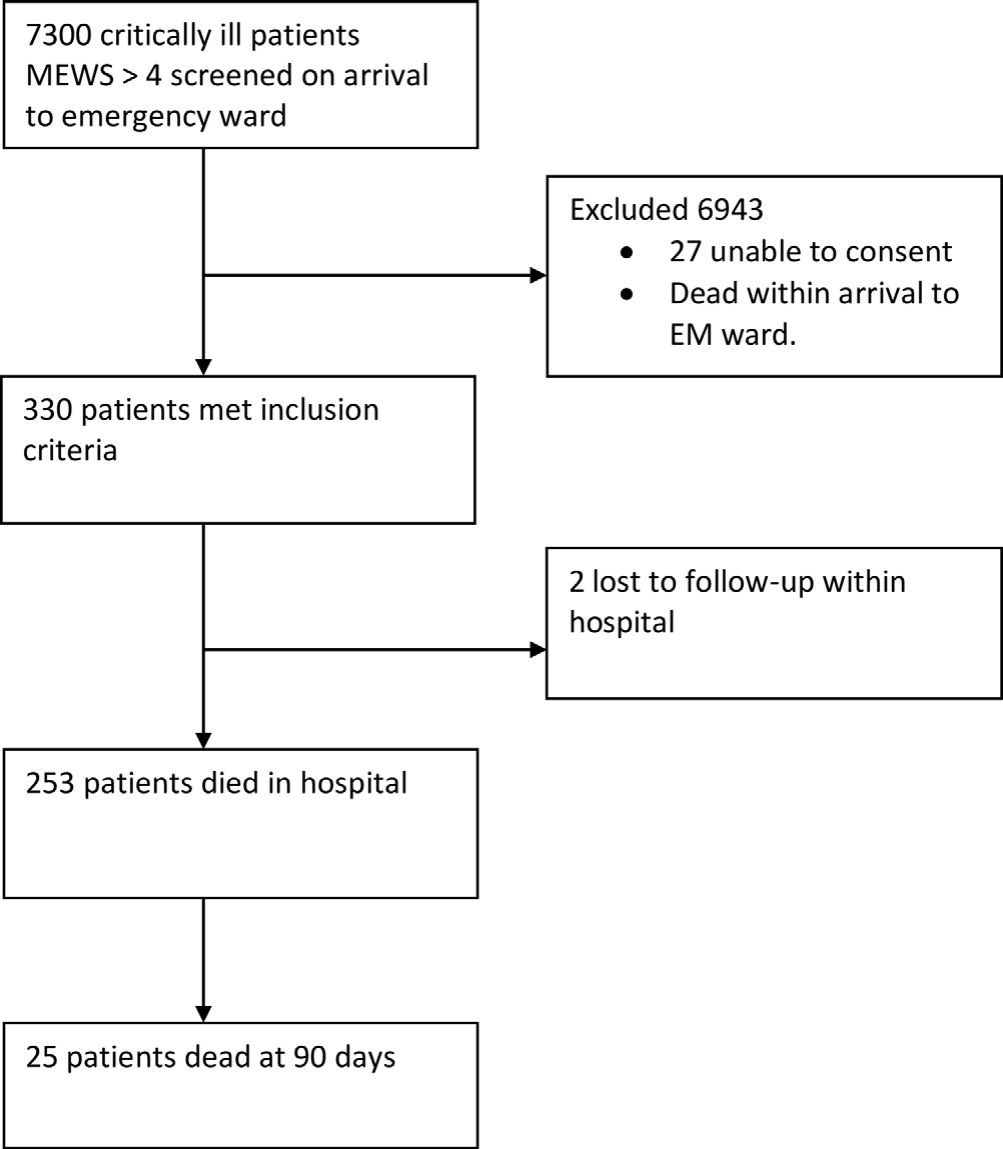
Flow chart of study participants. EM, Emergency Medicine; MEWS, Modified Early Warning Score.

We screened a total of 7300 acutely ill patients during the 18-month study period from 1 November 2015 to 31 July 2017. Of these, 327 patients met the eligibility criteria for AHRF. We excluded 6972 patients (figure 1). This represented a prevalence of acute respiratory failure of 4.5%. There was no difference between age and sex distribution at the screening and enrolment stages. The median age was 38 years (IQR 27–52) and the majority of the study patients (60%) was male (table 1). Of the 327 who presented with acute respiratory failure, 310 (94.5%) reported no previous history of acute respiratory failure. Cardiac disease was present in 28 (8.5%) and asthma in 6 (1.8%) study participants. The most common cause of AHRF was pneumonia among 271 (82.6%). Co-infection with HIV was present in 101 (30%) of the participants. The mean plethysmographic oxygen saturation by pulse oximetry (on room air) was 77.6% (SD 12.7); mean SpO_2_/FiO_2_ ratio 193.9 (SD 31.8); mean LIPS 6.7 (SD 0.8). Only 6% of the participants were admitted to the ICU where they received invasive mechanical ventilation.

**Table 1:** Baseline characteristics at admission

Characteristic	N (%)/327 (100)
	**Demographics**
Age in years: median (IQR)	38 (27,52)
Male	197 (60.1)
Use firewood	121 (36.8)
Smoking history	50 (15)
Smoking pack years*	0.95 (0.63)
History of asthma	6 (1.8)
Previous history of acute lung injury admission	18 (5.5)
	**Diagnosis at admission**
Pneumonia	271 (82.6)
Cardiac disease	28 (8.5)
Neurological disease	85 (25.9)
HIV-positive	101 (30.8)
Sepsis	100 (30.4)
	**Clinical characteristics†** **Mean (SD)**
Lung Injury Prediction Score	6.7 (0.8)
Heart rate	117.8 (22.8)
Respiratory rate	33.9 (8.9)
Mean arterial pressure (mm Hg)	78.6 (23.3)
Temperature (^o^C)	37.2 (1.1)
Pulse oximetry (SpO_2_)	77.6 (12.7)
SpO_2_/F ratio	193.9 (31.8)
White cell count (10^9^/L)‡	11.9 (8.3)
Haemoglobin status g/dL‡	11.1 (3.9)
Platelet count (10^9^/L)‡	243.2 (139.1)

*Pack years (SD).

†Worst values were recorded in the 24 hours.

‡While patients got a full blood count done free of charge, only 313/327 had Full Blood Count results available.

Regarding mortality (table 2), 253 (77%) of the study participants died in hospital while cumulative mortality at 90-day follow-up was 85%. Using modified Poisson regression analysis, severity of hypoxaemia (RR 1.29 (95% CI 1.15 to 1.54), p=0.01); a high LIPS (RR 1.79 (95% CI 1.14 to 2.83), p=0.01); thrombocytopenia (defined as <100) (RR 1.23 (95% CI 1.11 to 1.38), p=0.01); anaemia (RR 1.15 (95% CI 1.01 to 1.31), p=0.03); HIV-positive status (RR 0.84 (95% CI 0.72 to 0.97), p=0.019) and being male (RR 1.15 (95% CI 1.01 to 1.31), p=0.04) were independently associated with 90-day mortality (table 3). We found no statistically significant difference between mortality among those who received ICU care and those who did not. Unlike other studies, we did not find an association between older age and mortality.

**Table 2:** Bivariate analysis of factors associated with mortality

Characteristic	Outcome n (% row)		Crude risk ratio (95% CI)	P value
	**Dead**	**Alive**		
	254 (77.7)	73 (22.3)		
Sex				
Female	97 (74.6)	33 (25.4)	1	
Male	157 (79.7)	40 (20.3)	1.07 (0.94 to 1.21)	0.293
Previous history of acute lung injury admission				
No	240 (77.7)	69 (22.3)	1	
Yes	14 (77.8)	4 (22.2)	1.00 (0.78 to 1.29)	0.991
Smoked				
No	215 (77.6)	62 (22.4)	1	
Yes	39 (78.0)	11 (22.0)	1.00 (0.86 to 1.18)	0.952
Had asthma				
No	250 (77.9)	71 (22.1)	1	
Yes	4 (66.7)	2 (33.3)	0.86 (0.48 to 1.51)	0.593
Had pneumonia				
No	44 (77.2)	13 (22.8)	1	
Yes	210 (77.8)	60 (22.2)	1.01 (0.86 to 1.18)	0.924
Had a cardiac disease				
No	234 (78.3)	65 (21.7)	1	
Yes	20 (71.4)	8 (28.6)	0.91 (0.72 to 1.16)	0.459
Neuro				
Yes	186 (76.9)	56 (23.1)	1	
No	68 (80.0)	17 (20.0)	1.04 (0.92 to 1.18)	0.536
Liver				
No	245 (77.8)	70 (22.2)	1	
Yes	9 (75.0)	3 (25)	0.96 (0.69 to 1.34)	0.830
HIV status				
No	179 (79.2)	47 (20.8)	1	
Yes	75 (74.3)	26 (25.7)	0.94 (0.82 to 1.0.7)	0.341
	**Dead**	**Discharged**		
SPO_2_ /FiO_2_				
<200	139 (88.0)	19 (12.0)	1	
201 or more	115 (68.1)	54 (31.9)	0.77 (0.69 to 0.87)	<0.001
LIPS				
Low	14 (48.3)	15 (51.7)	1	
High (six or more)	240 (80.5)	58 (19.5)	1.67 (1.14 to 2.44)	0.009
Heart rate				
Normal	41 (74.6)	41 (25.4)	1	
Abnormal (>100)	205 (77.9)	58 (22.1)	1.05 (0.88 to 1.24)	0.602
Respiratory rate				
26 (81.3)	6 (18.7)	1	
74 (76.3)	23 (23.7)	0.94 (0.77 to 1.15)	0.538
154 (77.8)	44 (22.2)	0.96 (0.80 to 1.15)	0.639
MAP				
Normal	168 (72.4)	64 (27.6)	1	
Abnormal (<65)	86 (90.5)	9 (9.5)	1.25 (1.13 to 1.39)	<0.001
SPO_2_%				
Normal (85+)	77 (64.2)	43 (35.8)	1	
Abnormal	177 (85.5)	30 (14.5)	1.33 (1.15 to1.54)	<0.001
WBC×10 ul				
Normal	108 (68.3)	50 (31.7)	0.36 (0.13 to 0.98)	0.046
Abnormal (>12 000 or <4000)	126 (84.6)	23 (15.4)	0.89 (0.30 to 2.60)	0.830
Haemoglobin (/dl)				
Normal	176 (75.2)	58 (24.8)	1	
Abnormal (<8)	55 (83.3)	11 (16.7)	1.11 (0.97 to 1.26)	0.124
Platelet count (×10^9^/ul)				
Normal	166 (74.4)	57 (25.6)	1	
Abnormal (<100)	29 (96.7)	1 (3.3)	1.30 (1.17 to 1.44)	<0.001

MAP, mean arterial pressure.

**Table 3:** Multivariate analysis

Characteristic	Crude risk ratio (95% CI)	P value	Adjusted risk ratio (95% CI)	P value
Sex				
Female	1		1	
Male	1.07 (0.94 to 1.21)	0.293	1.15 (1.01 to 1.31)	0.044
HIV status				
No	1		1	
Yes	0.94 (0.82 to 1.0.7)	0.341	0.84 (0.72 to 0.97)	0.019
LIPS				
Low	1		1	
High (6 or more)	1.67 (1.14 to 2.44)	0.009	1.79 (1.14 to 2.83)	0.012
Heart rate				
Normal	1		1	
Abnormal (>100)	1.05 (0.88 to 1.24)	0.602	1.07 (0.89 to 1.29)	0.448
MAP				
Normal	1		1	
Abnormal (<65)	1.25 (1.13 to 1.39)	<0.001	1.09 (0.98 to 1.22)	0.125
SpO_2_%				
Normal (85+)	1		1	
Abnormal (<85)	1.33 (1.15 to 1.54)	<0.001	1.29 (1.10 to 1.51)	0.001
HBG/dl				
Normal	1		1	
Abnormal (<8)	1.11 (0.97 to 1.26)	0.124	1.15 (1.01 to 1.31)	0.028
Platelet count (×109/ul)				
Normal	1		1	
Abnormal (<100)	1.30 (1.17 to 1.44)	<0.001	1.23 (1.11 to 1.38)	<0.001

LIPS, Lung Injury Prediction Score; MAP, mean arterial pressure.

## Discussion

Our study showed that AHRF was prevalent and associated with a high mortality in our population. The risk factors for death were pneumonia, severity of hypoxaemia, high LIPS, HIV co-infection, anaemia, thrombocytopenia and being male.

We conducted a period prevalence study because considering the resource limitations it was operationally less complex as compared with the cohort or case-control study.

Our patients were predominantly male, of a similar age range as African studies, but much younger than patients in high-income countries (HICs).^9 10^

The prevalence of AHRF in our study population was low. This is slightly similar to a study done in Rwanda in which hypoxia was found in 8% of hospitalised patients. The Kigali Study screened 1046 hospitalised patients over a 6-week period. This was lower than that reported in the Scandinavian and LUNGSAFE studies which reported incidences of AHRF of 23% and 34%, respectively. However, the LUNGSAFE Study did have some middle-income country data.

The main cause of AHRF in our study population was lower respiratory tract infection (LRTI). The majority of our study participants had pneumonia as a diagnosis. Community-acquired bacterial pneumonia was the most common cause of ARDS in data from other studies in the African setting.^11^

These findings concur with data suggesting that a young adult with LRTI in an LIC has a much higher odds of dying compared with one with LRTI in a high-income country.^2^ Similar findings were reported in the Rwanda Study where the most common underlying cause of respiratory failure was infection. Our study however, found that none of the participants was assessed for microbiological aetiology.

Our study found mortality to be very high among patients with AHRF. This was higher than that reported in the Kigali Study. This was even much higher than that reported in both the Scandinavian and the LUNGSAFE (Large observational study to Understand the Global Impact of Severe Acute Respiratory failure) studies, even though in these studies, mortality was almost identical between the patients with non-ARDS acute respiratory failure and patients with mild or moderate ARDS.^2 4^Mortality among our patients was higher than the overall mortality among adult patients admitted to the Mulago National Referral and Teaching Hospital with non-respiratory acute illness (from an audit conducted at the same time, the mortality rate for non-AHRF acutely ill patients was 48%).

The high mortality in our study may be explained by the fact that our patients were very sick. This may be evidenced by the fact that LIPS was very high; that patients were severely hypoxaemic at presentation; and that due to resource constraints (such as lack of ICU beds, fluid monitoring, etc) most of our patients who would have benefited from intensive care, were not able to access it.

LIPS has been used to identify patients at risk of developing ARDS. In that study, a cut-off of ≥4 predicted having ARDS or death.^7^ In our study, the average LIPS was more than 2 points above that cut-off. LIPS maybe useful in helping identify those high-risk patients in whom low cost interventions would possibly save, given the limited and often expensive treatment options available once ARDS has developed.

Severity of hypoxaemia is a predictor of mortality among patients with ARDS.^12^ Severe hypoxaemia (PaO_2_/FiO_2_ < 100 mm Hg, at a PEEP (Positive End Expiratory Pressure) of 5 cm H_2_O), has been found in 20%–30% of patients with ARDS and is associated with the highest mortality rate.^1 13^ The mean non-invasive SpO_2_ in our study was 77%. Additionally, we also used the SpO_2_/FiO_2_ ratio to determine the severity of hypoxaemia. This is now a widely accepted non-invasive method to determine severity of hypoxaemia in hypoxic acute lung disease.^8 14 15^ The mean SpO_2_/FiO2 ratio (193.9) in our study suggests that the majority of our study patients, and using the AECC (American European Consensus Conference) definitions,^16^ had acute lung injury (ALI).

HIV co-infection was associated with higher mortality from respiratory failure, as seen in previous studies in Uganda.^17 18^ The immunosuppressive state is known to predispose to aggressive opportunistic infections that cause respiratory and other organ failures. The significance of the association between anaemia and thrombocytopenia with mortality in our study explains the high rate of infection and possible pulmonary sepsis. In sepsis, a low platelet count is a well-known biomarker for disease severity.^19^ This (in addition to hypotension) highlights the high incidence of sepsis in this study population. Hypotension was also a predictor of mortality among patients with pneumonia in a study conducted in Malawi.^20^ We did not employ sepsis identification strategies at the time of the study. The focus was on treatment of infection and not necessarily sepsis as a syndrome. A limitation in our study was we did not document how much fluid was administered to patients. A randomised fluid trial in Zambia was stopped early for possible harm in the subgroup of patients with hypoxaemia and tachypnoea (who were receiving fluids).^21^ It is possible that in our study population, fluids given to treat hypotension caused more harm. However, hypotension was not significantly associated with mortality at multivariate analysis.

Another possible explanation for the high mortality in our study was the lack of adequate resources to provide positive pressure ventilation to all patients that would benefit from it. Only 6% of the study participants received mechanical ventilation. Even then, mortality among them was still high. Some data suggest that mechanically ventilated patients in LICs have a higher mortality than those in HICs.^22^ Other resource constraints such as staffing, skills and delays in accessing care may explain this.

The Rwanda Study used a modification of the Berlin definition to achieve their primary objective.^6^ They found that about 50% of all patients with an SpO_2_/FiO_2_ ratio ≤315 (where their estimate of PaO_2_/FiO_2_ ≤300) met their criteria for ARDS. Both the LUNGSAFE Study and a study looking at the impact of high-flow oxygen in severe respiratory failure found that the majority of hypoxic patients met the criteria for ARDS.^2 23^ Therefore, while our study was not able to apply the Berlin definition, it may seem logical that a large percentage of our hypoxic patients would have met clinical criteria for ARDS.

Our study had some limitations. Due to resource constraints, as previously documented, we were unable to perform arterial blood gas measurements in the study population. However, as documented earlier, we used a validated method to detect severity of hypoxaemia. We were unable to perform microbiological diagnoses such as blood cultures or sputum analyses. Other investigations were sporadic and inconsistent. We relied on clinicians’ judgement for diagnosis data, but the ward system ensured that an internal medicine specialist had reviewed the working diagnosis. Another likely limitation is that we conducted a single-centre study. However, being a national referral hospital, the study site receives patients from all over the country.

Our study is probably the first of its kind in Africa to explore the prevalence and outcomes of AHRF in an adult population in a limited resource acute care setting.

The prevalence of AHRF among emergency department patients in a tertiary hospital in an LIC was low but was associated with very high mortality. Pneumonia was the most common cause of AHRF. Severity of HIV co-infection, hypoxaemia, high LIPS, anaemia, thrombocytopenia and being male were independently associated with death. This highlights the need for more work on early detection and management of AHRF in resource-limited settings.
